# Effects of Gastric Acid Secretion Inhibitors for Ventilator-Associated Pneumonia

**DOI:** 10.3389/fphar.2022.898422

**Published:** 2022-05-05

**Authors:** Fang Li, Hui Liu, Luming Zhang, Xiaxuan Huang, Yu Liu, Boen Li, Chao Xu, Jun Lyu, Haiyan Yin

**Affiliations:** ^1^ Department of Intensive Care Unit, The First Affiliated Hospital of Jinan University, Guangdong, China; ^2^ Department of Intensive Care Unit, The Second Affiliated Hospital of Bengbu Medical College, Bengbu, China; ^3^ Department of Neurology, The First Affiliated Hospital of Jinan University, Guangdong, China; ^4^ Department of Clinical Research, The First Affiliated Hospital of Jinan University, Guangdong, China; ^5^ Guangdong Provincial Key Laboratory of Traditional Chinese Medicine Informatization, Guangdong, China

**Keywords:** ventilator-associated pneumonia, mortality, proton pump inhibitor, histamine 2 receptor antagonist, gastric acid secretion inhibitors

## Abstract

**Objective:** This study analyzed the association of gastric acid secretion inhibitors (GASIs) [including proton pump inhibitors (PPIs) and histamine 2 receptor antagonists (H2RAs)] with the occurrence of ventilator-associated pneumonia (VAP) and in-hospital mortality in patients who received invasive mechanical ventilation (IMV).

**Method:** Patients who received IMV and used GASI were included based on records in the MIMIC-IV database. The relationships of GASIs with VAP and the in-hospital mortality were determined using univariate and multivariate logistic regression analyses. Also, the effects of GASIs in some subgroups of the population were further analyzed.

**Results:** A total of 18,669 patients were enrolled, including 9191 patients on H2RAs only, 6921 patients on PPIs only, and 2557 were on a combination of the two drugs. Applying logistic regression to the univariate and multivariate models revealed that compared with H2RAs, PPIs had no significant effect on the incidence of VAP, and the combination of H2RAs and PPIs was a risk factor for VAP. Compared with H2RAs, univariate logistic regression revealed that, PPIs and combine the two drugs were both risk factors for in-hospital mortality, but multivariate logistic regression showed that they were not significantly associated with in-hospital mortality. In subgroup analysis, there were interaction in different subgroups of age, PCO2, myocardial infarct, congestive heart failure (P for interaction<0.05).

**Conclusion:** Compared with H2RAs, PPIs did not have a significant association with either VAP or in-hospital mortality; the combination of H2RAs and PPIs was risk factor for VAP, but did not have a significantly associated with in-hospital mortality.

## Introduction

Invasive mechanical ventilation (IMV) is commonly used in critically ill patients in intensive care units (ICUs) to maintain airway patency, prevent aspiration, and improve oxygenation. A critical complication is ventilator-associated pneumonia (VAP). Patients who received IMV had pulmonary infections mostly caused by pathogens present in the hospital environment (Timsit et al., 1000). A meta-analysis estimated that the incidence density of VAP/1000 ventilator days was 15.1%, with higher incidence rates in low-income countries (Bonell et al., 2019). VAP increases hospital stays, mortality, and treatment costs (Koenig and Truwit, 2006; Fadda and Ahmad, 2022).

Patients with IMV are often associated with stress ulcer risk, and preventive application of gastric acid secretion inhibitors (GASIs) can significantly reduce gastrointestinal bleeding (Zhou et al., 2019). The most commonly used drugs are proton pump inhibitors (PPIs) and histamine 2 receptor antagonists (H2RAs). H2RAs were thought to inhibit gastric acid secretion by blocking histamine receptors, PPIs work by inhibiting the final stages of gastric acid production (Toews et al., 2018). On the one hand, these drugs can improve the pH gastric juice and allow excessive bacterial growth (Buendgens et al., 2016); on the other hand, they can reduce reflux, and prevent bacteria movement to the pharynx and lungs. The purpose of this study was to determine the effects of two different GASIs on VAP incidence and in-hospital mortality among patients with IMV.

## Methods

### Data Source

The Beth Israel Deaconess Medical Center (BIDMC), Massachusetts General Hospital, and the Massachusetts Institute of Technology jointly developed the Medical Information Mart for Intensive Care (MIMIC) in 2003. The project was funded by the National Institutes of Health (Yang et al., 2020). The MIMIC-IV database provides information on more than 70,000 patients admitted to the ICU of BIDMC from 2008 to 2019, and it is freely available to researchers. All patients were re-identification under the Safe harbor provisions of and Health Insurance Portability and Accountability Act, thereby waiving the need to obtain informed consent (Wu et al., 2021).

### Patient Population

Patients were searched for in the MIMIC-IV database. The inclusion criteria identified 25,031 patients who used IMV. The exclusion criteria were 1) not the first ICU admission (2,495 patients), 2) ICU stay of less than 24 h (1379 patients), 3) >5% missing data (141 patients), and 4) not used PPIs or H2RAs (2444 patients).

### Variable Selection and Outcome

Baseline characteristics during ICU admission were obtained using Structured Query Language, and if there were multiple values, the worst values for that period were recorded. Information was collected on demographic characteristics (year of admission, sex, body weight, and age), disease severity [Acute Physiology Score III (APSIII), Sequential Organ Failure Assessment scores (SOFA), laboratory examination results white blood cells (WBC), hemoglobin (HB), platelets (PLT)], [partial pressure of carbon dioxide (PCO2), partial pressure of oxygen (PO2), lactate, anion gap, blood urea nitrogen (BUN), blood sugar (GLU), international normalized ratio (INR), and total bilirubin (TBIL)], commodities (myocardial infarction, congestive heart failure, peptic ulcer disease, diabetes, cerebrovascular disease, renal disease, liver disease, and sepsis), treatment regimens [renal replacement therapy (RRT) vasopressors use, PPIs, H2RAs, and IMV duration], hospital length of stay (LOS), and ICU LOS. PPIs included esomeprazole, lansoprazole, omeprazole, and pantoprazole; and H2RAs included famotidine, ranitidine, and cimetidine.

End points were a VAP diagnosis and all-cause in-hospital mortality. According to the standard VAP diagnostic criteria of the Centers for Disease Control and Prevention, the specific diagnostic criteria used in this study are listed in Table 1 (American Thoracic Society and Infectious Diseases Society of America, 2005; Klompas et al., 2012).

**TABLE 1:** Centers for Disease Control and Prevention’s clinical surveillance definition for VAP.

Category	Detailed Description
Radiologic criteria (two or more serial radiographs with at least one of the following)	1. New or progressive and persistent infiltrate
	2. Consolidation
	3. Cavitation
Systemic criteria (at least one)	1. Fever (>38 °C or > 100.4°F)
	2. Leukopenia (<4000 WBC/mm3) or leukocytosis (≥12,000 WBC/mm3)
	3. For adults ≥70 years old, altered mental status with no other recognized cause
Pulmonary criteria (at least two)	1. New onset of purulent sputum, or change in character of sputum, or increased respiratory secretions, or increased suctioning requirements
	2. Worsening gas exchange (e.g., desaturations, increased oxygen requirements, or increased ventilator demand)
	3. New onset or worsening cough, or dyspnea, or tachypnea
	4. Rales or bronchial breath sounds

All patients on ventilation more than 48 h.

### Statistical Analysis

Data were collated and outliers were deleted. If a variable had >20% missing data, it was discarded; otherwise, the 10-degree interpolation method was applied. Categorical data were expressed as frequencies or percentages. Continuous variables were expressed as medians and interquartile ranges. Chi-square and Kruskal–Wallis H tests were used to identify significant differences between the groups.

Logistic regression was used to analyze the relationships of PPIs, H2RAs, and the combination of PPIs and H2RAs with VAP and in-hospital mortality. These results were expressed as odds ratios (ORs) and 95% confidence intervals (CIs). The logistic regression presupposes a linear relationship between the continuous independent variables and logit(P), so we first performed the boxTidwell test (Brescia et al., 2021).*p* values for the variables age, weight, PLT, AG, INR, and TBIL were all greater than 0.05 (Supplementary Table S1) consistent with a linear relationship with logit(P), and the remaining continuous variables were converted to categorical variables based on clinical significance or inter-quartile spacing. Univariate and multivariate models were established for each end point. In the multivariate model, we adjusted for patients’ general information age, sex, weight; disease severity score APSIII, SOFA score; routine hematological tests WBC, HB, PLT; patients’ liver and kidney functions: BUN, TBIL, INR; indicators reflecting patients’ internal environment PO2, PCO2, anion gap, GLU, lactate and patients’ major comorbidities myocardial infarction, congestive heart failure, cerebrovascular disease, peptic ulcer disease, liver disease, diabetes, renal disease, sepsis were adjusted. In addition, whether patients were on RRT after ICU admission, vasopressors use, and IMV duration were also used for adjustment. The variance inflation factor showed no multicollinearity between these variables (Supplementary Table S2).

The relationships of PPIs, H2RAs, and the combination of PPIs and H2RAs with VAP were assessed using subgroup analyses. The subgroups were classified according to age, sex, APSIII, SOFA score, PO2, PCO2, HB, WBC, PLT, anion gap, BUN, INR, body weight, GLU, TBIL, lactate, myocardial infarct, congestive heart failure, cerebrovascular disease, peptic ulcer disease, liver disease, diabetes, renal disease, sepsis, RRT, vasopressors use, and IMV duration.

R software (http://www.R-project.org) and SPSS software (version 27.0, IBM, United States) were used for all statistical analyses. All probability values were bilateral, and *p* < 0.05 was considered indicative of statistical significance.

## Results

### Clinical Characteristics

**FIGURE 1:**
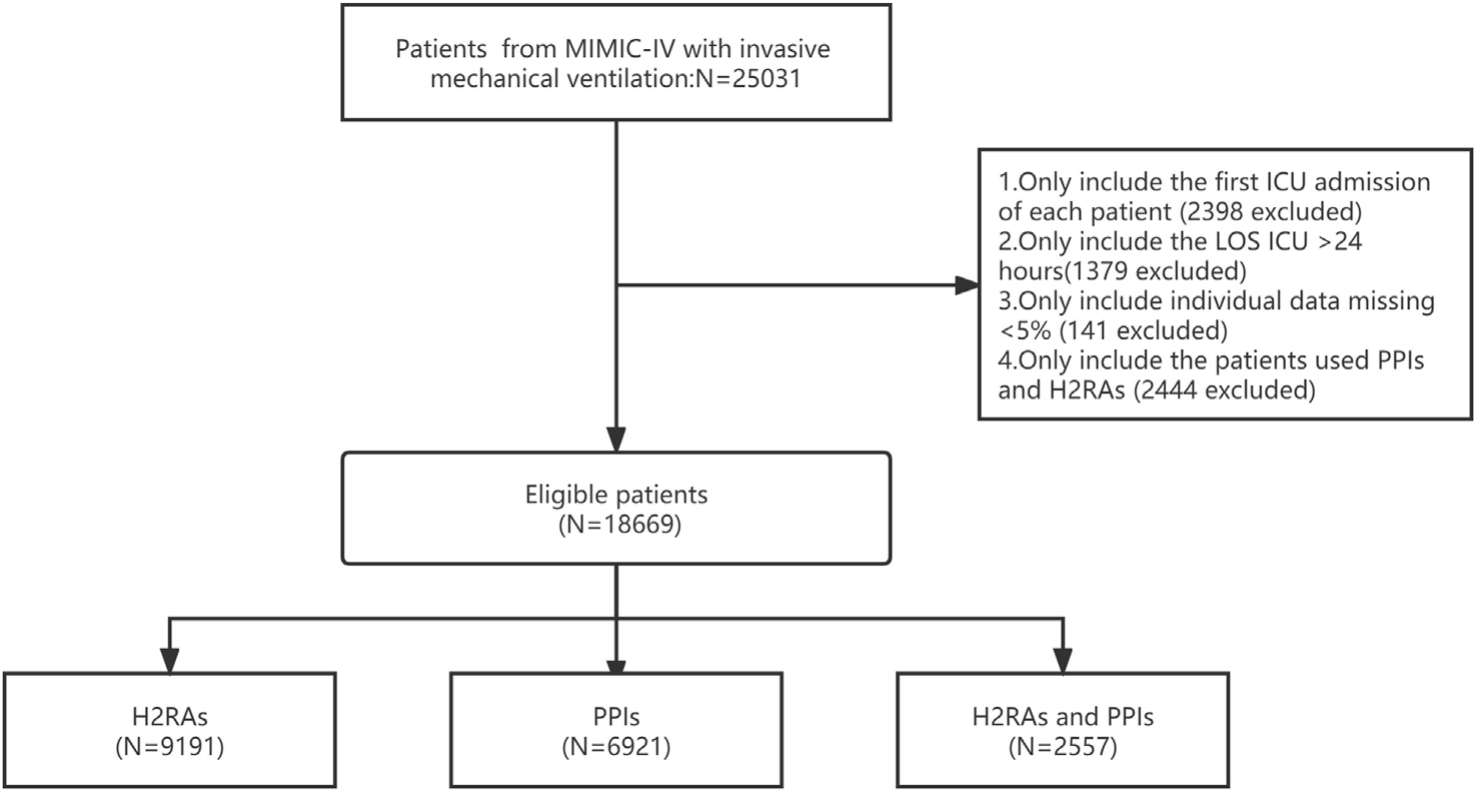
Illustration of exclusion and inclusion criteria.

This study enrolled 18,669 ICU patients with IMV and GASIs (see flowchart in Figure 1). There were 9191 patients in the H2RAs group, 6,921 patients in the PPIs group, 2,557 patients in the combination of PPIs and H2RAs group. H2RAs group were more male (*p* < 0.05). PPIs group were older (*p* < 0.05). The combination of PPIs and H2RAs group had higher APSIII, SOFA scores; more vasopressors and RRT use; longer IMV duration, hospital LOS and ICU LOS (*p* < 0.05). In comorbidity, patients in the three groups were most complicated with sepsis (67.7%, 76.2% and 84.8%, respectively).More detailed characteristics of the remaining participants stratified by different GASIs can be seen in Table 2.

**TABLE 2:** Characteristics of patients with GASIs.

	H2RAs	PPIs	H2RAs and PPIs	*p*
N	9191	6921	2557	—
Age (year)	66.00 (54.00, 76.00)	67.00 (56.00, 77.00)	66.00 (55.00, 77.00)	<0.001
Sex	—	—	—	<0.001
** **Male	5634 (61.3)	4009 (57.9)	1465 (57.3)	—
** **Female	3557 (38.7)	2912 (42.1)	1092 (42.7)	—
Weight (kg)	82.40 (69.60, 98.05)	80.97 (68.30, 96.70)	83.28 (70.20, 98.53)	<0.001
Year of admission	—	—	—	<0.001
** **2008–2010	2805 (30.5)	2680 (38.7)	943 (36.9)	—
** **2011–2013	2092 (22.8)	1597 (23.1)	571 (22.3)	—
** **2014–2016	2322 (25.2)	1551 (22.4)	582 (22.8)	—
** **2017–2019	1972 (21.5)	1093 (15.8)	461 (18.0)	—
APSIII	—	—	—	<0.001
** **<58	6225 (67.7)	3496 (50.5)	994 (38.9)	—
** **58–82	1848 (20.1)	1822 (26.3)	763 (29.8)	—
** **>82	1118 (12.2)	1603 (23.2)	800 (31.3)	—
SOFA	—	—	—	<0.001
** **<6	3489 (38.0)	1858 (26.8)	446 (17.4)	—
** **6–9	3910 (42.5)	2519 (36.4)	911 (35.6)	—
** **>9	1792 (19.5)	2544 (36.8)	1200 (46.9)	—
Vasopressors	—	—	—	<0.001
** **No	8178 (89.0)	5719 (82.6)	1882 (73.6)	—
** **Yes	1013 (11.0)	1202 (17.4)	675 (26.4)	—
RRT	—	—	—	<0.001
** **No	8842 (96.2)	6171 (89.2)	2148 (84.0)	—
** **Yes	349 (3.8)	750 (10.8)	409 (16.0)	—
IMV duration (hour)	—	—	—	<0.001
** **<96	6694 (72.8)	4495 (64.9)	1012 (39.6)	—
** **≥96	2497 (27.2)	2426 (35.1)	1545 (60.4)	—
WBC(K/uL)	—	—	—	<0.001
** **<10	993 (10.8)	1025 (14.8)	191 (7.5)	—
** **≥10	8198 (89.2)	5896 (85.2)	2366 (92.5)	—
HB(g/L)	—	—	—	<0.001
** **<120	8624 (93.8)	6692 (96.7)	2487 (97.3)	—
** **≥120	567 (6.2)	229 (3.3)	70 (2.7)	—
PLT (K/uL)	132.00 (99.00, 179.00)	123.00 (73.00, 181.00)	114.00 (62.00, 171.00)	<0.001
INR	1.40 (1.20, 1.80)	1.50 (1.30, 2.30)	1.60 (1.30, 2.70)	<0.001
Anion gap (mmol/L)	17.00 (15.00, 19.00)	18.00 (15.00, 22.00)	19.00 (16.00, 23.00)	<0.001
TBIL (mg/dL)	0.60 (0.40, 1.10)	0.80 (0.50, 2.10)	0.90 (0.50, 2.30)	<0.001
PO2(mmHg)	—	—	—	<0.001
** **<60	3135 (34.1)	3591 (51.9)	1562 (61.1)	—
** **≥60	6056 (65.9)	3330 (48.1)	995 (38.9)	—
PCO2(mmHg)	—	—	—	<0.001
** **<50	4907 (53.4)	3327 (48.1)	883 (34.5)	—
** **≥50	4284 (46.6)	3594 (51.9)	1674 (65.5)	—
PH	—	—	—	<0.001
** **<7.35	6263 (68.1)	4978 (71.9)	2074 (81.1)	—
** **≥7.35	2928 (31.9)	1943 (28.1)	483 (18.9)	—
Lactate (mmol/l)	—	—	—	<0.001
** **<3.3	6396 (69.6)	4528 (65.4)	1338 (52.3)	—
** **≥3.3	2795 (30.4)	2393 (34.6)	1219 (47.7)	—
BUN(mg/dl)	—	—	—	<0.001
** **<20	2544 (27.7)	1228 (17.7)	343 (13.4)	—
** **≥20	6647 (72.3)	5693 (82.3)	2214 (86.6)	—
Glucose (mg/dl)	—	—	—	<0.001
** **<200	6075 (66.1)	3912 (56.5)	1192 (46.6)	—
** **≥200	3116 (33.9)	3009 (43.5)	1365 (53.4)	—
Myocardial infarct	—	—	—	0.074
** **No	7495 (81.5)	5570 (80.5)	2041 (79.8)	—
** **Yes	1696 (18.5)	1351 (19.5)	516 (20.2)	—
Congestive heart failure	—	—	—	<0.001
** **No	6862 (74.7)	4650 (67.2)	1697 (66.4)	—
** **Yes	2329 (25.3)	2271 (32.8)	860 (33.6)	—
Cerebrovascular disease	—	—	—	<0.001
** **No	7398 (80.5)	6042 (87.3)	2053 (80.3)	—
** **Yes	1793 (19.5)	879 (12.7)	504 (19.7)	—
Peptic ulcer disease	—	—	—	<0.001
** **No	9132 (99.4)	6469 (93.5)	2468 (96.5)	—
** **Yes	59 (0.6)	452 (6.5)	89 (3.5)	—
Liver disease	—	—	—	<0.001
** **No	8480 (92.3)	5417 (78.3)	2143 (83.8)	—
** **Yes	711 (7.7)	1504 (21.7)	414 (16.2)	—
Diabetes	—	—	—	<0.001
** **No	6544 (71.2)	4653 (67.2)	1742 (68.1)	—
** **Yes	2647 (28.8)	2268 (32.8)	815 (31.9)	—
Renal disease	—	—	—	<0.001
** **No	7683 (83.6)	5211 (75.3)	1926 (75.3)	—
** **Yes	1508 (16.4)	1710 (24.7)	631 (24.7)	—
Sepsis	—	—	—	<0.001
** **No	2966 (32.3)	1644 (23.8)	388 (15.2)	—
** **Yes	6225 (67.7)	5277 (76.2)	2169 (84.8)	—
VAP	—	—	—	<0.001
** **No	8590 (93.5)	6432 (92.9)	2132 (83.4)	—
** **Yes	601 (6.5)	489 (7.1)	425 (16.6)	—
In-hospital mortality	—	—	—	<0.001
** **No	7904 (86.0)	5383 (77.8)	1886 (73.8)	—
** **Yes	1287 (14.0)	1538 (22.2)	671 (26.2)	—
LOS ICU (day)	3.46 (2.03, 7.04)	4.04 (2.21, 7.92)	7.75 (3.71, 15.08)	<0.001
LOS hospital (day)	8.87 (5.62, 14.88)	9.88 (5.95, 16.82)	15.95 (8.80, 26.96)	<0.001

*p* < 0.05 means significant difference.

**FIGURE 2:**
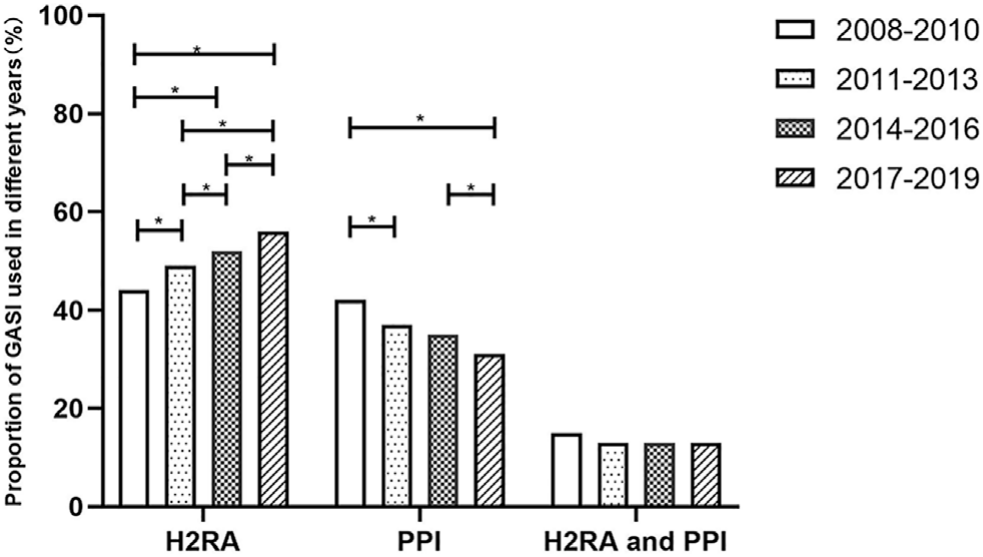
Comparison of distribution proportion between GASIs in different years.

The distribution of GASIs in different years was shown in Figure 2. The proportion of H2RAs increased year by year, and the comparison among all groups was significant, and the highest in 2017–2019 group (55.93%) (*p* < 0.05). The proportion of PPIs decreased year by year, except that there was no statistical difference between 2011–2013 group and 2014–2016 group (*p* > 0.05), other groups had statistical differences, the lowest in 2017–2019 group (31.00%) (*p* < 0.05). There was no statistical difference in the combination of PPIs and H2RAs among all groups (*p* > 0.05).

### Relationships of GASIs With VAP and In-Hospital Mortality

Applying logistic regression revealed that compared with H2RAs group, the combination of PPIs and H2RAs group was a risk factor for VAP in the univariate and multivariate models (*p* < 0.05), whereas PPIs group was not significantly associated with VAP (*p* > 0.05). Compared with H2RAs, univariate logistic regression revealed that, PPIs and the combination of H2RAs and PPIs were both risk factors for in-hospital mortality, but multivariate logistic regression revealed that PPIs and the combination of H2RAs and PPIs were not significantly associated with in-hospital mortality (*p* > 0.05) (Table 3).

**TABLE 3:** Logistic regression analysis of GASIs to VAP and in hospital mortality for both models.

—		Univariate Model		Multivariate Medol	
		OR [CI(95%)]	*p* Value	OR [CI(95%)]	*p* Value
VAP	H2RAs	reference	—	reference	—
	PPIs	1.087 [0.960-1.229]	0.188	0.883 [0.769-1.014]	0.08
	H2RAs and PPIs	2.849 [2.493-3.253]	<0.001	1.476 [1.273-1.711]	<0.001
In-hospital mortality	H2RAs	reference	—	reference	—
	PPIs	1.755 [1.617-1.904]	<0.001	1.105 [0.997-1.224]	0.06
	H2RAs and PPIs	2.185 [1.965-2.429]	<0.001	0.903 [0.792-1.028]	0.12

OR, odds ratio; CI: confidence interval. *p* < 0.05 means significant difference.

Multivariate logistic regression, variables: age, sex, weight, APSIII, SOFA, WBC, HB, PLT, PCO2, PO2, BUN, INR, TBIL, lactate, glucose, anion gap, congestive heart failure, cerebrovascular disease, peptic ulcer disease, myocardial infarct, liver disease, diabetes, renal disease, sepsis, vasopressors, RRT, IMV, duration.

### Subgroup Analysis

Subgroup analyses were performed on various covariate, most of which presented no obvious interaction (Table 4, *p* > 0.05). There were interactions in different subgroups of age, PCO2, myocardial infarct, congestive heart failure (P for interaction<0.05). PPIs was a protect factor for VAP in the subgroup of age <65 years, APSIII <58, PCO2<50 mmHg, IMV duration≥96 h, without used vasopressors, without myocardial infarct, without congestive heart failure, without liver disease, without diabetes, without renal disease. (*p* < 0.05).

**TABLE 4:** Subgroup analysis of the associations between GASIs and VAP.

	H2RAs		PPIs		H2RAs and PPIs		P For Interaction
			OR [CI(95%)]	*p* Value	OR [CI(95%)]	*p* Value	
Age (years)	—	—	—	—	—	—	0.048
** **<65	8522	reference	0.730 (0.597–0.892)	0.026	1.299 (1.050–1.604)	<0.001	—
** **≥65	10147	reference	1.046 (0.862–1.269)	0.650	1.702 (1.380–2.097)	<0.001	—
Gender	—	—	—	—	—	—	0.782
** **Male	11108	reference	0.900 (0.755–1.072)	0.237	1.420 (1.173–1.717)	<0.001	—
** **Female	7561	reference	0.862 (0.686–1.081)	0.200	1.608 (1.266–2.038)	<0.001	—
SOFA	—	—	—	—	—	—	0.156
** **<6	5793	reference	0.720 (0.504–1.017)	0.066	1.422 (0.929–2.135)	0.097	—
** **6–9	7340	reference	0.831 (0.667–1.033)	0.097	1.496 (1.182–1.888)	0.001	—
** **>9	5536	reference	1.053 (0.845–1.314)	0.648	1.601 (1.275–2.013)	<0.001	—
Vasopressors	—	—	—	—	—	—	0.092
** **NO	15779	reference	0.839 (0.718–0.979)	0.026	1.375 (1.158–1.631)	<0.001	—
** **YES	2890	reference	1.127 (0.816–1.564)	0.470	1.861 (1.356–2.568)	<0.001	—
CRRT	—	—	—	—	—	—	0.896
** **NO	17161	reference	0.905 (0.779–1.050)	0.187	1.506 (1.281–1.768)	<0.001	—
** **YES	1508	reference	0.809 (0.547–1.204)	0.291	1.364 (0.914–2.049)	0.131	—
Sepsis	—	—	—	—	—	—	0.051
** **NO	4998	reference	0.953 (0.452–1.957)	0.897	3.337 (1.551–6.968)	0.002	—
** **YES	13671	reference	0.873 (0.758–1.005)	0.060	1.426 (1.226–1.658)	<0.001	—
Myocardial infarct	—	—	—	—	—	—	0.001
** **NO	15106	reference	0.810 (0.694–0.945)	0.008	1.518 (1.290–1.786)	<0.001	—
** **YES	3563	reference	1.231 (0.891–1.706)	0.210	1.376 (0.950–1.989)	0.090	—
Congestive heart failure	—	—	—	—	—	—	0.047
** **NO	13209	reference	0.792 (0.666–0.942)	0.009	1.475 (1.231–1.766)	<0.001	—
** **YES	5460	reference	1.091 (0.863–1.382)	0.468	1.517 (1.164–1.976)	0.002	—
Cerebrovascular disease	—	—	—	—	—	—	0.282
** **NO	15493	reference	0.914 (0.781–1.069)	0.260	1.474 (1.239–1.752)	<0.001	—
** **YES	3176	reference	0.800 (0.589–1.081)	0.150	1.542 (1.153–2.057)	0.003	—
Peptic ulcer disease	—	—	—	—	—	—	0.061
** **NO	18069	reference	0.885 (0.769–1.018)	0.087	1.454 (1.250–1.689)	<0.001	—
** **YES	600	reference	3.036 (0.712–22.018)	0.187	7.474 (1.681–55.389)	0.019	—
Liver disease	—	—	—	—	—	—	0.272
** **NO	16040	reference	0.845 (0.725–0.983)	0.030	1.470 (1.251–1.726)	<0.001	—
** **YES	2629	reference	1.182 (0.828–1.703)	0.363	1.762 (1.177–2.651)	0.006	—
Diabetes	—	—	—	—	—	—	0.163
** **NO	12939	reference	0.820 (0.694–0.969)	0.020	1.378 (1.152–1.646)	<0.001	—
** **YES	5730	reference	1.040 (0.811–1.335)	0.755	1.763 (1.346–2.308)	<0.001	—
Renal disease	—	—	—	—	—	—	0.063
** **NO	14820	reference	0.825 (0.703–0.967)	0.018	1.473 (1.245–1.741)	<0.001	—
** **YES	3849	reference	1.173 (0.878–1.573)	0.284	1.652 (1.192–2.289)	0.003	—
APSIII	—	—	—	—	—	—	0.132
** **<58	10715	reference	0.771 (0.605–0.979)	0.034	1.719 (1.319–2.228)	<0.001	—
** **58–82	4433	reference	0.972 (0.768–1.228)	0.810	1.485 (1.155–1.907)	0.002	—
** **>82	3521	reference	0.888 (0.689–1.146)	0.359	1.322 (1.016–1.721)	0.038	—
WBC (K/uL)	—	—	—	—	—	—	0.472
** **<10	2209	reference	0.625 (0.360–1.078)	0.092	0.950 (0.427–1.976)	0.895	—
** **≥10	16460	reference	0.899 (0.779–1.037)	0.144	1.501 (1.289–1.746)	<0.001	—
HB (g/L)	—	—	—	—	—	—	0.902
** **<120	17803	reference	0.886 (0.771–1.018)	0.089	1.480 (1.275–1.718)	<0.001	—
** **≥120	866	reference	0.594 (0.135–2.195)	0.456	1.694 (0.275–7.966)	0.530	—
PCO2 (mmHg)	—	—	—	—	—	—	0.004
** **<50	9117	reference	0.662 (0.518–0.842)	0.001	1.445 (1.106–1.879)	0.006	—
** **≥50	9552	reference	1.027 (0.865–1.219)	0.758	1.524 (1.272–1.825)	<0.001	—
PO2 (mmHg)	—	—	—	—	—	—	0.497
** **<60	8288	reference	0.924 (0.782–1.093)	0.359	1.508 (1.261–1.804)	<0.001	—
** **≥60	10381	reference	0.796 (0.616–1.022)	0.077	1.433 (1.088–1.874)	0.009	—
Lactate (mmol/L)	—	—	—	—	—	—	0.189
** **<3.3	12262	reference	0.879 (0.741–1.042)	0.138	1.356 (1.112–1.650)	0.002	—
** **≥3.3	6407	reference	0.921 (0.724–1.171)	0.501	1.618 (1.281–2.045)	<0.001	—
Glucose (mg/dl)	—	—	—	—	—	—	0.879
** **<200	11179	reference	0.905 (0.734–1.114)	0.347	1.481 (1.171–1.868)	0.001	—
** **≥200	7490	reference	0.862 (0.716–1.037)	0.116	1.475 (1.215–1.789)	<0.001	—
BUN (mg/dl)	—	—	—	—	—	—	0.944
** **<20	4115	reference	0.871 (0.557–1.340)	0.534	1.324 (0.780–2.187)	0.285	—
** **≥20	14554	reference	0.884 (0.764–1.023)	0.098	1.485 (1.271–1.735)	<0.001	—
IMV duration (hour)	—	—	—	—	—	—	0.196
** **<96	12201	reference	0.942 (0.722–1.227)	0.661	1.780 (1.256–2.485)	0.001	—
** **≥96	6468	reference	0.846 (0.719–0.994)	0.043	1.403 (1.190–1.654)	<0.001	—

OR, odds ratio; CI: confidence interval. *p* < 0.05 means significant difference.

Multivariate logistic regression, variables: age, sex, weight, APSIII, SOFA, WBC, HB, PLT, PCO2, PO2, BUN, INR, TBIL, lactate, glucose, anion gap, congestive heart failure, cerebrovascular disease, peptic ulcer disease, myocardial infarct, liver disease, diabetes, renal disease, sepsis, vasopressors, RRT, IMV, duration.

## Discussion

The patients who received IMV in the ICU had severe diseases that were often accompanied by stress ulcers, resulting in inadequate nutritional supplies, and further digestive tract hemorrhage, gastrointestinal perforation, and life-threatening conditions in serious cases. Stress ulcer treatment guidelines for gastrointestinal bleeding prevention in critically ill patients recommend using PPIs (or H2RAs as a reasonable alternative), but sucralfate is not recommended (Ye et al., 2020). Previous research has linked stress ulcer medication use to an increased VAP risk. Our current study showed that 18,669 patients had received GASIs while on IMV therapy, and 1,515 (8.12%) of them developed VAP, which is a relatively high rate. Moreover, we found that the utilization rate of H2RAs increased year by year compared with PPIs, and the proportion of patients using only H2RAs reached 55.93% from 2017 to 2019. To investigate the prognostic impact of specific GASIs, this study investigated the effect of PPI, H2RAs and their combination on VAP and in-hospital mortality. The results showed that compared to H2RAs, PPIs was found no significant effect on VAP or in-hospital mortality, but their combination was risk factors for VAP whereas no significant effect on in-hospital mortality.

Aspiration is an important factor in hospital acquired pneumonia (HAP) and VAP pathogenesis. By inhibiting acid production, PPIs and H2RAs increase nosocomial pathogen colonization in the oropharynx and trachea, while delaying gastric emptying, greatly increasing the risk of infection from aspiration (Tablan et al., 2004). The flora analysis of VAP also supported this, and the pathogenic bacteria of patients who received PPIs and H2RAs were mostly *pseudomonas*, Gram-negative bacteria, and methicillin-resistant *Staphylococcus aureus*, which may be caused by gastric alkalinization (Grindlinger et al., 2016). In critically ill patients, there was no difference in pneumonia incidence between H2RAs and PPIs, but PPIs significantly reduced GIB incidence (Deliwala et al., 2021), and H2RAs had a lower cost and higher survival rate (MacLaren and Campbell, 2014). Both of them have advantages and disadvantages. However, some studies have suggested the presence of a difference in VAP incidence between the two GASIs. Miano et al. reported that pantoprazole was associated with a higher HAP incidence than was ranitidine (Miano et al., 2009). A meta-analysis also found that PPIs are more effective than H2RAs, but may also increase the risk of VAP (Alquraini et al., 2017). Zhou et al. believed that for high-risk critically ill patients, PPIs and H2RAs can significantly reduce gastrointestinal bleeding, with both potentially increasing the pneumonia incidence (Zhou et al., 2019). Other studies had shown that there was no difference in the effect of PPI on VAP compared with H2RAs, but PPI had a better anti-gastrointestinal bleeding effect (Arriola et al., 2016). Some studies had pointed out that PPIs and H2RAs had no effect on the risk of death or extubation in critically ill patients (Li et al., 2020). The effect of PPIs and H2RAs on VAP, the results of previous studies were inconsistent.

In this study, PPIs compared with H2RAs had no difference in VAP and in hospital mortality. The combination of PPIs and H2RAs would lead to a higher incidence of VAP, but did not have a beneficial effect on in hospital mortality, which reminds us not to use the two drugs to inhibit gastric acid secretion at the same time. We hypothesized that the combination of the two drugs meant that the dose was increased, the inhibition of gastric acid secretion was stronger, and the gastric alkalization was more serious. On the one hand, the incidence of VAP was increased, and on the other hand, the treatment effect of stress ulcer was better, thus having the impact on mortality. Further confirmation is certainly needed.

This study found that PPIs compared with H2RAs was a protect factor for VAP in subgroup of age<65 years, APSIII <58, PCO2<50 mmHg, without used vasopressors, without myocardial infarct, without congestive heart failure, without liver disease, without diabetes, without renal disease. The effect of PPIs on VAP was more beneficial in mild, young patients without chronic heart, renal and liver disease. Therefore, we still suggest that more consideration should be given to the use of PPIs after the evaluation of patients with IMV, and PPIs was also the preferred drug for stress ulcer in the guidelines. Studies have shown that compared with the using H2RAs, PPIs prophylaxis was the most effective preventive strategy in patients at high risk of developing stress ulcer bleeding (Barkun et al., 2013). However, in recent years, the proportion of H2RAs had increased rapidly, and the reasons affecting doctors’ decision-making need to be further discussed.

In clinical practice, it is necessary to measure VAP incidence and the efficiency of stress ulcer prevention and treatment, and select appropriate drugs according to the situation of each patient. In this study, the combination of PPIs and H2RAs is not recommended, which increases the risk of VAP. In patients with less severe disease, younger and with fewer comorbidities, PPIs was associated with a lower incidence of VAP compared to H2RAs. However, no matter how GASIs was selected, it had no significant impact on in hospital mortality.

### Limitations

This study analyzed a big-data sample spanning 12 years, but there were some limitations. First, it uses a retrospective single-center design, which has some uncontrolled confounding bias. Second, there was no comparison of the effects of the drugs on gastrointestinal bleeding to determine their therapeutic effects. Third, logistic regression presupposes a linear relationship between continuous independent variables and logit(P), but in some machine learning methods such as ensemble modeling, non-linearity can be handled automatically without prior specification, and the application of this method can be tried in future research (Zhang et al., 2022). Finally, due to some limitations in the database, the total dose and duration of the drug could not be accurately calculated in this study, and these factors could be taken into account in the design of future studies.

## Conclusion

Compared with H2RAs, PPIs did not have a significant association with either VAP or in-hospital mortality; the combination of H2RAs and PPIs was risk factor for VAP, but did not have a significantly associated with in-hospital mortality.

## Abbreviation

APSIII, Acute physiology score III; BUN, Blood Urea Nitrogen; GASIs, Gastric acid secretion inhibitors; H2RAs, Histamine 2 receptor antagonist; INR, international normalized ratio; IMV, Invasive mechanical ventilation; PPIs: Proton pump inhibitors; PO2, Partial pressure of blood oxygen; PCO2, Partial Pressure of Carbon Dioxide; RRT, Renal replacement therapy; SOFA, Sequential organ failure assessment; VAP, ventilator-associated pneumonia; WBC, White Blood Cell.

## Data Availability

The datasets presented in this study can be found in online repositories. The names of the repository/repositories and accession number(s) can be found below: The data were available on the MIMIC-IV website at https://mimic-iv.mit.edu.
